# Supplementary material for: Smokers’ cognitive and behavioural reactions during the early phase of the COVID-19 pandemic: Findings from the 2020 ITC Four Country Smoking and Vaping Survey
Source: PLoS One. 2021 Jun 4;16(6):e0252427. doi: 10.1371/journal.pone.0252427 (PMC8177641; doi:10.1371/journal.pone.0252427)
Supplement: Attachment — Submitted filename: Response to Reviewers.docx [file pone.0252427.s001.docx]

**Responses to Reviewer 1:**

**Reviewer #1: This is a well-written manuscript showing findings from a cross-sectional study conducted on a sample of tobacco smokers on the potential impact of the coronavirus pandemic on smoking habit during the early phase of the COVID-19 era. Data are original, the issue is interesting and the conclusions are acceptable. Authors should consider the following minor points to improve the presentation of findings.**

1. **Please save room in the Abstract to clarify the level of the Covid-19 lockdown during the fieldwork in various countries.**

Authors’ Response: We have added a brief summary in the abstract. However, readers can refer to Figure 1 for details about restrictions/lockdowns in each country. The following was added to the methods section of the abstract:

Methods: *“…These four countries had varying responses to the pandemic by governments and public health, ranging from advising voluntary social distancing to implementing national and subnational staged lockdowns. Considering these varying responses, and the differences in the number of confirmed cases and deaths (greatest in England and the US and lowest in Australia), smoking behaviours related to COVID-19 may have differed between countries…”*

2. **This study has some limitations, partially addressed in the Discussion section. Although the ITC study is a longitudinal study, at the end within this manuscript Authors considered a cross-sectional analysis, only. Thus, this analysis has the limitations inherent to the cross-sectional studies.**

Authors’ Response: We agree that cross-sectional studies do have inherent methodological limitations. However, because COVID-19 is a new pandemic, we do not have previous studies to compare responses. We have added the following to the limitations section to clarify this issue.

Page 25: *“…First, this study was cross-sectional and cannot be used to infer causality. However, the survey questions assessing thoughts about quitting and related changes in smoking were intentionally very specific, therefore we have no reason to believe that those who endorsed COVID-19 as a reason for having thoughts about quitting, reducing smoking, or making a quit attempt were not directly related to COVID-19….”*

3. **Authors “excluded the small number of ex-smokers who quit smoking during COVID-19”. It is not clear to me if this has been due to the fact that the study did not allow Authors to identify all subjects who quit smoking due to the COVID-19 pandemic. In any case, these analyses do not allow Authors to answer to the following research question: did the Covid-19 pandemic reduce or increase smoking prevalence and consumption? In my opinion, this is a limitation of this study.**

Authors’ Response: Although the survey did not allow us to confirm the precise date when recent smokers had quit, we did ask all recent quitters (those who quit smoking within the last two years, N=1010) if they had quit smoking because of COVID-19. In response to this question, only 58 respondents reported that they had quit because of COVID, 10 refused to answer this question, and 31 did not know. The question was not applicable to the remaining respondents because they had likely quit before the pandemic (or for other reasons). Thus, due to this small sample size, we could not include them in the models due to power issues. We explain this by this addition to the Limitations section:

Page 26*: “…Fifth, we were unable to include those who quit smoking because of the pandemic, due to the small number of those who quit reporting this (n=58) (many of the recent ex-smokers herein quit prior to the pandemic), thus the estimate for reduced smoking/making a quit attempt is a slight underestimate of respondents who did so….”*

**Responses to Reviewer 2:**

**Reviewer #2: This study examines changes in and reasons for changes in smoking and vaping during the COVID-19 pandemic across four countries. There are several issues with the paper, including those related to novelty, measures, and analyses.

Introduction**

1. **The introduction needs focus and a stronger literature review. There are many studies examining changes in smoking behavior during the pandemic, and a few studies have examined changes in vaping. While these are reviewed, the review is brief and the addition that the current study brings other than across countries isn’t developed well. For example, even just indicating that few studies have examined changes in vaping should be stated (although there are some more recent studies that should be included). The expanse of variables included in studies examining changes in tobacco use during the pandemic isn’t stated as should be. For example, some studies have focused on changes in smoking at all, with many findings within the same publication often showing some smokers increasing, some decreasing and some showing no changes at all. Other studies have shown changes in the number or frequency of use. These findings should be reviewed more in the current paper.**

Authors’ Response: At the initial time of writing the introduction for this paper, there were only a handful of published studies. Since then, and because of the duration of the review period, several more papers have been published. We have thus revised the introduction to include a more expansive summary of the current literature on smoking risks and COVID-19 (pages 4/5) and smoking behavior during the pandemic (pages 5/6). We have, however, refrained from commenting on changes in vaping, as this is beyond the scope of this study (we only evaluated changes in smoking as our outcome in this study). The impact of the pandemic on vaping will be explored in a future paper.

In addition to adding an updated literature summary, we have expanded on the reasons why our paper is important and novel (pages6/7). For example, we have conducted a more in-depth summary than the majority of existing publications on the relationship between smoking and COVID-19. We have noted that there are many methodological shortcomings in the available research that have examined factors related to changes in smoking (e.g., most do not include an evaluation of perceptions of personal susceptibility and severity of COVID-19 related to smoking, most do not present a conceptual model that identifies and tests the association of other variables with smoking that may be related to changes in smoking). And thus we conclude from our more extensive literature review, that more rigorous and robust studies are warranted (page 6).

Our study presents a much more substantial evaluation of the impact of the COVID-19 pandemic across a large and broad representative sample of smokers in four countries, all of which received identical survey questions, thus allowing a more accurate analysis across various countries. Most other studies that have made cross-country comparisons do so with results that are not fully comparable across countries due to differing methodologies.

Our survey questions also directly asked if their smoking changed because of COVID-19, which many studies only examined whether there were changes during the pandemic, and not explicitly because of the pandemic. Overall, our paper reports on a more extensive evaluation—both in the richness of the measures, defined by our conceptual model, and in the cross-country comparisons—than any previous study. Further, our creation and application of the conceptual model underlying our study will have heuristic value for developing a richer understanding both the extent to which, and the possible mechanisms for HOW the COVID-19 pandemic has affected smoking behaviours.

2. **The paper examines factors associated with changes in smoking and vaping during the pandemic. There are several studies that have included and examined reasons for changes in smoking and vaping, including risk perceptions. These studies are not discussed in the paper, nor are the reasons for this paper articulated.**

Authors’ Response: As mentioned above, our study included an examination of what factors were associated with changes in smoking only. Changes in vaping will be addressed in a separate paper. We have added in a more thorough narrative summary of the literature related to changes in smoking during COVID-19 into the Introduction (as stated above). This content was also discussed previously in the Discussion section. This added content begins on page 4. The updated paragraph that outlines studies that have examined risk perceptions can be found on page 6.

3. **The first paragraph of the introduction, while interesting, seems unnecessary and better for the discussion if at all.**

Authors’ Response: We agree, and have therefore removed this paragraph from the Introduction. We have also slightly revised the beginning of the Introduction to support the flow after removing this initial paragraph.

4. **Similarly, much of the discussion in the introduction concerning reasons for including the 4 countries is more of a commentary and better belongs in the discussion. For example, describing that England was overwhelmed or messages being misaligned don’t belong in the introduction. Instead, simply indicating that the 4 countries had different responses and differing messages might contribute to differing perceptions and tobacco use behaviors is all that is needed.**

Authors’ Response: We agree, and have removed most of this content from the Introduction in the last paragraph. We have kept in a little of the content to provide background and clarity as to why smokers across the four countries may have differed in behavioural smoking changes related to COVID-19.

5. **The introduction offers a hypothesis earlier in the introduction before the research questions are offered, and a second hypothesis later in the introduction. Further, there are 3 supplemental analyses that are interesting and should be included as part of the main questions (especially iii, which is really related to the Health Belief Model).**

Authors’ Response: We have re-organized the last paragraph of the introduction to reflect this suggestion. Our main hypothesis is now at the end of the paragraph (after the study aims). However, while we recognize that all of the study objectives are important, our primary objective was to explore if the pandemic increased the likelihood that smokers would think about quitting and motivate behavior change toward quitting, including making quit attempts, or at least reducing their consumption.

Since recent findings suggest that smoking may worsen COVID-19 outcomes—a suggestion that has been highlighted in the national health authorities of all four of our countries—we directly addressed the research question of whether smokers would motivated to reduce or stop their smoking as a consequence of this messaging. We feel that our rationale is warranted since it reflects such an important question. With regard to the supplemental analyses, we did however want to add these other analyses in order to provide a more complete picture (e.g., what characteristics were related to increased smoking), which is why they were initially classified as supplemental. However, upon reflection, we agree with the reviewer that we should bring those supplemental analyses into the main analyses. Accordingly, we now outline the three primary objectives of the study in the abstract and the introduction, and we no longer refer to the additional analyses as “supplemental”. The aims have been re-worded and are followed by our main hypothesis (page 7):

*The primary aims of this study were to examine the association between COVID-19 and: (1) thoughts about quitting smoking; (2) changes in smoking behaviour (quit attempts, reduced or increased smoking, or no change in smoking); and (3) factors related to a positive change (making a quit attempt or reducing smoking) based on an adapted framework of the HBM. We also examined: (4) what personal modifying factors (e.g., sociodemographics, mental health, nicotine dependence) were related to increased smoking because of COVID-19; (5) whether there were country differences among smokers and worry about getting COVID-19; and (6) whether those who tried to quit in the past month attributed their quit attempt to COVID-19. We hypothesized that because Australia was least affected, possibly owing to both being geographically isolated and the implementation of an early coordinated response by national, regional, and local governments that included a national lockdown, public adherence to the rules, and widespread testing [49], smokers in Australia would show lower levels of smoking-related cognitive and behavioural changes relative to smokers in the other three countries.*

6. **Would be good to update the number of COVID-19 cases and deaths given that the numbers change so quickly**

Authors’ Response: We have updated the cases and deaths in the introduction (as of April 8, 2021). We will continue to do so up to our final submission since, disturbingly, these vital statistics are rapidly increasing from day to day.

**Methods**

1. **The survey items are not well developed and worded. For example, “How worried are you that you already have, or that you will get the coronavirus” is “double-barreled” and might result in unreliable responses. People who already have COVID-19 might not be worried especially if they are not that sick; whereas those who don’t have it but are concerned/at-risk might be more worried.**

Authors’ Response: In the original version of this manuscript, we stated in the Statistical Analyses section that respondents who had already received a positive COVID-19 test at the time of data collection (n=218) were excluded from the analytical models, because the questions ‘worried they already had or would get COVID-19’ and ‘if you got the coronavirus, how severe do you think the illness would be for you, compared to others your age who got it’ were not relevant. We have added this information to the Measures section now as well for clarity (page 9). These 218 respondents were included in the basic evaluations of the two outcomes (Tables 2 and 3), but were not included in the larger analytical models. We do not expect that the few respondents who had already tested positive during the initial wave of the pandemic would not have made a difference to the results.

2. **The outcome variable concerning thinking about quitting during the pandemic is also problematic, as the survey didn’t first ask about desire to quit before asking if a reason to want to quit has to do with the virus. It is possible that people were heading towards quit anyway.**

Authors’ Response: To clarify this query, the survey did include a general question about intentions to quit smoking. This was then followed up about reasons why they were thinking about quitting using separate questions (In the past 6 months, have any of the following things led you to think about quitting?). The reasons asked were: concern for your personal health, the price of cigarettes, and the coronavirus outbreak. The response options for each question included: Not at all, Somewhat, Very much, Don't know. Therefore the outcome variable about the pandemic causing respondents to think about quitting is by design, very specific. Our objective in doing this was that if the respondent thought about quitting for any of these reasons, they would select ‘very much’ or ‘somewhat’. And while this study was cross-sectional and cannot be used to infer causality (for example, intentions to quit indeed could have preceded the pandemic), we have no reason to believe that those who endorsed COVID-19 as a reason for having thoughts about quitting were not directly related to COVID-19, as opposed to some other reason. We have added this as a limitation as also suggested by Reviewer 1 (see page 25).

3. **Also, including “don’t know” with “I didn’t think about it…” is an assumption that probably should not be made. Similar issues are seen with some of the other survey questions. Granted, the items cannot be changed, but these issues should be acknowledged.**

Authors’ Response: Our categorization into “yes” vs. “no/don’t know” has been re-worded into “yes” vs. “otherwise”. Our objective for this analysis was to explore the positive responses (Yes: very much or somewhat), and “don’t know” does not qualify as a positive response. We have updated this in the statistical analyses section and under Tables 4 and 5.

**Results**

4. **In the first set of results, regarding thoughts about quitting, the narrative only discusses 3 of the 4 countries. The data for the US should be stated too.**

Authors’ Response: We have added the following text to the results (page 14): “…The US did not significantly differ from Australia (43.3%, p=0.10), but US smokers were less likely to have thought about quitting than those in Canada (p=0.03) and England (p=0.003)”.

5. **Unclear why the variables in the supplemental analyses are supplemental and not part of the main analyses? Understanding why people increased is also very important.**

Authors’ Response: As noted in the response above, we have removed reference to the supplemental analyses. Notably, while our intentions were to examine positive changes in smoking because of COVD-19, we recognize that the original supplemental analyses are also warranted, in order to more fully explain the varied reactions.

6. **The results don’t separate out changes in vaping versus smoking. Are there differences worth noting?**

Authors’ Response: As noted in the responses above, we did not evaluate changes in vaping. Vaping status was a covariate in the larger models as a modifying factor (Tables 3 and 4). We felt this was appropriate, as dual users may have decreased smoking or tried to quit relative to non-vapers, as they would have had some form of nicotine substitution. However, while current vapers were more likely to think about quitting smoking relative to non-vapers (and may be vaping with plans for quitting smoking), there was no difference in reducing/quitting smoking because of COVID-19 between these two groups. Additionally, as this paper is already lengthy, a future paper will evaluate changes in vaping during COVID.

**Discussion**

7. **The main differences in this study compared to the many other studies examining changes in tobacco use during the pandemic have to do with the vaping findings and the 4 countries. However, very little of the discussion focuses on these more novel aspects of the data.**

Authors’ Response: This paper did not evaluate changes in vaping (as noted above), although we concede that this evaluation by country is warranted and will be applied in a subsequent paper.

**Figures**

8. **Don’t think Figure 2 is necessary**

Authors’ Response: We agree, as these measures have been described in the Methods section already, thus, we have removed Figure 2 (pictorial of the Health Belief Model).
